# Predictive Value of FOLFOX-Based Regimen, Long Interval, Hemoglobin Levels and Clinical Negative Nodal Status, and Postchemoradiotherapy CEA Levels for Pathological Complete Response in Patients with Locally Advanced Rectal Cancer after Neoadjuvant Chemoradiotherapy

**DOI:** 10.1155/2020/9437684

**Published:** 2020-01-28

**Authors:** Chun-Ming Huang, Ching-Wen Huang, Cheng-Jen Ma, Yung-Sung Yeh, Wei-Chih Su, Tsung-Kun Chang, Hsiang-Lin Tsai, Suh-Hang Juo, Ming-Yii Huang, Jaw-Yuan Wang

**Affiliations:** ^1^Department of Radiation Oncology, Kaohsiung Municipal Ta-Tung Hospital, Kaohsiung Medical University, Kaohsiung, Taiwan; ^2^Department of Radiation Oncology, Kaohsiung Medical University Hospital, Kaohsiung Medical University, Kaohsiung, Taiwan; ^3^Graduate Institute of Medicine, College of Medicine, Kaohsiung Medical University, Kaohsiung, Taiwan; ^4^Department of Radiation Oncology, Faculty of Medicine, College of Medicine, Kaohsiung Medical University, Kaohsiung, Taiwan; ^5^Division of Colorectal Surgery, Department of Surgery, Kaohsiung Medical University Hospital, Kaohsiung Medical University, Kaohsiung, Taiwan; ^6^Department of Surgery, Faculty of Medicine, College of Medicine, Kaohsiung Medical University, Kaohsiung, Taiwan; ^7^Graduate Institute of Clinical Medicine, College of Medicine, Kaohsiung Medical University, Kaohsiung, Taiwan; ^8^Division of Trauma and Critical Care, Department of Surgery, Kaohsiung Medical University Hospital, Kaohsiung Medical University, Kaohsiung, Taiwan; ^9^Center for Cancer Research, Kaohsiung Medical University, Kaohsiung, Taiwan

## Abstract

We aimed to identify predictors of a pathological complete response (pCR) in patients with locally advanced rectal cancer (LARC) following a multimodality therapy. We retrospectively reviewed 236 patients with LARC treated with neoadjuvant chemoradiotherapy (CRT) followed by radical resection from January 2011 to December 2017. Patients were administered CRT, which comprised radiotherapy and chemotherapy with an oxaliplatin plus 5-fluorouracil- or fluoropyrimidine-based regimen. Clinical factors were correlated with treatment response. The multivariate logistic regression revealed that a negative nodal stage (odds ratio (OR) = 3.2, *P*=0.0135), a high hemoglobin level (>10 g/dL) during neoadjuvant CRT (OR = 3.067, *P*=0.0125), an oxaliplatin-containing neoadjuvant CRT (OR = 5.385, *P*=0.0044), a long interval (>8 weeks) between radiotherapy and surgery (OR = 1.135, *P*=0.0469), and a post-CRT CEA ≤2 ng/mL (OR = 2.891, *P*=0.0233) were the independent predictors of increased pCR rates. The prediction nomogram was developed according to the above independent variables. The concordance index was 0.74, and the calibration curve showed good agreement. In summary, negative nodal stages, high hemoglobin levels during treatment, oxaliplatin-containing neoadjuvant therapy, a long radiotherapy-surgery interval (>8 weeks), and post-CRT CEA levels ≤2 ng/mL were favorable predictors of a pCR. This prediction nomogram might be crucial for patients with LARC undergoing a multimodality therapy.

## 1. Introduction

For locally advanced rectal cancer (LARC), neoadjuvant chemoradiotherapy (CRT) has been the standard treatment because it provides high local control, low treatment toxicity, high rates of sphincter preservation, and improved disease-free survival (DFS) [1–3]. However, the literature shows inconsistent treatment response to neoadjuvant CRT, ranging from a pathological complete response (pCR) to total resistance. A pCR to neoadjuvant CRT has been associated with a low rate of recurrence and favorable survival, but the incidence of pCR has ranged from 10% to 30% [2–8]. Therefore, how to identify patients who can benefit the most from neoadjuvant CRT remains unresolved.

Although numerous methods to predict a pCR before surgery have been investigated, the predictors identified are not consistent across studies [9, 10]. Some clinical parameters and molecular biomarkers have been reported to be predictors of a pCR to neoadjuvant CRT for patients with LARC [9–11]. Several clinical factors, such as pretreatment T or N stage, serum carcinoembryonic antigen (CEA), chemotherapy regimen, and radiation dose, have been associated with a pCR [5,12–16]. Few studies have investigated the effect of treatment-related hematologic toxicity on a pCR. Therefore, we aimed to identify a correlation between hematologic toxicity with pCR in this study.

Identifying the predictive factors of pCR is helpful because of the efficacy and safety of watch-and-wait approaches for patients who achieved a pCR to neoadjuvant CRT [17]. The benefit of watch-and-wait approaches is the avoidance of a morbid radical resection without compromising tumor control [18]. The dilemma we encountered is how to identify the patients who might be benefited by watch-and-wait approaches. The aim of this study was to identify the predictive factors of pCR in patients with LARC who may benefit from watch-and-wait strategies.

## 2. Materials and Methods

### 2.1. Patients

We analyzed 248 patients with histopathologically proven locally advanced rectal adenocarcinoma (T3-4 or N+) who received neoadjuvant CRT followed by total mesorectal excision (TME) in a single institute from January 2011 to December 2017. The exclusion criteria involved previous or synchronous malignancies other than nonmelanoma skin cancer, local excision of rectal tumor, and a history of pelvic irradiation. Twelve patients were excluded from this analysis because of incomplete neoadjuvant CRT (*n* = 4), refusal of surgery (*n* = 3), unresectable tumors at surgery (*n* = 3), and local excision of primary tumor only (*n* = 2). The remaining 236 LARC patients without evidence of distant metastasis were enrolled. Our institutional review board approved this study. Pretreatment evaluation included a medical history review, physical examination, colonoscopy with tumor biopsy, chest radiography, abdominal computed tomography (CT), pelvic magnetic resonance imaging (MRI), a serum CEA assessment, and routine laboratory studies.

### 2.2. Chemotherapy

A fluoropyrimidine-based regimen was delivered to 95 patients. The regimen included (1) 5-fluorouracil (5-FU; 350 mg/m^2^, intravenous bolus) and leucovorin (20 mg/m^2^, intravenous bolus) on days 1 to 5 and days 21 to 25 of irradiation, once every 2 weeks or (2) 6 cycles of capecitabine 850 mg/m^2^ twice daily for 14 days, followed by 7 days of rest after each cycle [19]. For the rest of the 141 patients, a biweekly schedule of FOLFOX was prescribed. Each cycle of FOLFOX consisted of oxaliplatin (85 mg/m^2^) on day 1, folinic acid (400 mg/m^2^), and a 46-h infusion of 5-FU (2800 mg/m^2^) repeated every 2 weeks. After irradiation completion, all patients continued biweekly FOLFOX until 2 to 3 weeks before surgery.

### 2.3. Radiotherapy

Patients were simulated with CT in a supine position with a customized thermoplastic immobilization device. All patients were instructed to void their bladder and then drink 300 ml of water 30 min before simulation and irradiation. The total radiation dose was delivered in a range of 45 to 50.4 Gy using a daily fraction of 1.8 to 2.0 Gy. We added a 1.5 to 2 cm clinical target margin to cover the gross tumor volume. Beyond the clinical target margin, we added a planning target margin of 1 to 1.5 cm. All patients received external-beam radiotherapy with either 3-dimensional conformal or intensity-modulated radiation therapy.

### 2.4. Surgery and Pathology Review

All patients underwent TME after neoadjuvant CRT completion [19]. There were 207 patients (87.7%) undergoing low anterior resection with colorectal or coloanal anastomosis and 29 patients (12.3%) receiving abdominoperineal resection. Two experienced pathologists, who are specialized in colorectal cancer, assessed the tumor response to neoadjuvant CRT. A pCR was defined as the absence of any viable cancer cells in the primary tumor and nodes (ypT0N0) in resected specimens after neoadjuvant treatment. More than 10% of mucins in resection specimens were considered as the presence of mucin pools and lacking neoplastic epithelium in specimens was defined as acellular mucin [20].

### 2.5. Evaluation and Follow-Up

During neoadjuvant CRT, acute toxicities were evaluated at each visit according to the Common Terminology Criteria for Adverse Events (CTCAE), version 4.03. In this study, anemia was defined as hemoglobin (Hb) level <10 g/dL. We evaluated post-CRT response approximately 6–10 weeks after completion of CRT. Digital rectal examinations, colonoscopy, CEA test, abdominal and chest CT, and pelvic MRI were used for post-CRT clinical assessment. MRI was used for evaluation of post-CRT locoregional stage, and CT was used for assessment of distant metastasis. Cancer restaging was recorded according to the 7th edition American Joint Committee on Cancer staging system. Post-CRT ycT0 stage was defined as no tumor in the rectal wall; ycT1 stage was defined as a tumor confined to the submucosa; ycT2 stage was defined as a tumor that had invaded the muscularis propria; and ycN stage 0 was defined as the absence of metastatic lymph nodes. Using T2-weighted MR images, a low to intermediate signal-intensity lesion was considered as a residual tumor; a metastatic lymph node was considered as a size threshold of >5 mm in short axis with heterogeneous signal or ill-defined margins.

After surgery, patients visited the outpatient department every 3 months in the initial 2 years and then once every 6 months to date. We defined tumor recurrence within the pelvis as a local failure and outside the pelvis as a distant failure.

### 2.6. Statistics

Fisher's exact test was used to compare categorical data between patients with and without a pCR. Multivariate logistic regressions were used to analyze variables with a *P* value <0.2 in univariate analysis to hope for adjustments of potential colinearity. A prediction nomogram was built to show the predicted probabilities of a pCR rate using variables with a *P* value <0.05 in multivariate analysis. A score of each independent predictive factor can be read out, and the sum of scores was converted to a probability of pCR. A bootstrap validation method was used for internal validation of a prediction nomogram. A total of 118 bootstrap samples were randomly selected, and the concordance index (C-index) was generated to estimate the bias-corrected or overfitting-corrected predictive discriminative ability of the model.

DFS was measured from the date of starting neoadjuvant CRT to the date of any type of recurrence or last follow-up. Overall survival (OS) was defined as the time from the start of neoadjuvant CRT to death from any cause or to last follow-up. Kaplan–Meier methods were used for DFS and OS, and the log-rank test was used to compare time-to-event distributions. Data analyses were performed using the SAS version 9.3 (SAS Institute, Cary, NC) and Stata version 14. A *P* value <0.05 was considered significant.

## 3. Results

The median age of the 236 patients was 63 years (range, 34–93 years). The majority of the patients had a clinical T3 primary tumor (77.9%) and clinical nodal metastasis (84.7%). Most patients received a total radiation dose of 50 Gy in 25 fractions (75.8%). Forty-five patients were treated with three-dimensional conformal radiation therapy, and 191 patients were treated with intensity-modulated radiation therapy. The median number of cycles of chemotherapy was seven (range, 5–9), with 80 patients (33.9%) receiving at least seven cycles of chemotherapy. The median number of post-CRT CEA was 2.2 ng/mL, ranging from 0.48 to 197.5 ng/mL. Therefore, 2 ng/mL was selected as the cutoff value for post-CRT CEA level.  Table 1 summarizes the characteristics of patients with and without a pCR. In the retrospective cohort, 56 patients (23.7%) achieved a pCR. Among the pCR patients, 12 patients (21.4%) were found to have acellular mucin pools. There was no local or distant recurrence in pCR patients with acellular mucin and two pCR patients without mucin developed tumor recurrence. Univariate analysis revealed that a negative nodal stage (*P*=0.0202), CRT with FOLFOX-based chemotherapy (*P*=0.0412), high Hb levels (>10 g/dL) during neoadjuvant CRT (*P*=0.0159), long interval (>8 weeks) between radiotherapy and surgery (*P*=0.0081), and post-CRT CEA levels ≤2 ng/mL (*P*=0.0285) were favorable predictors of a pCR.

**Figure 1:**
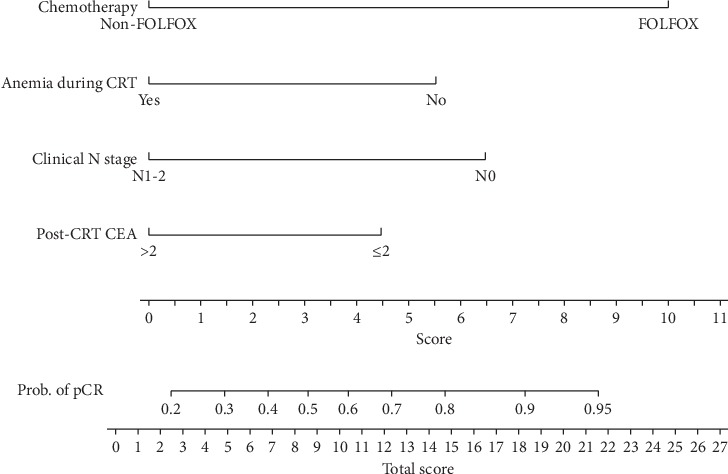
Nomogram developed for prediction of pathological complete response (pCR) rates. A score of each predictive factor can be read out at the top scale, and the sum of scores is converted to a probability of pCR. CEA, carcinoembryonic antigen; CRT, chemoradiation therapy; pCR, pathological complete response.

**Figure 2:**
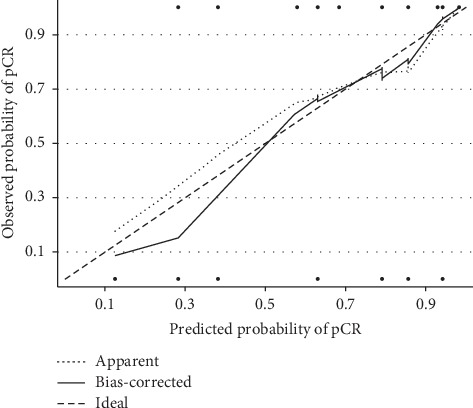
Calibration curve of observed and predicted probabilities. The *x* axis is the predicted probabilities measured by the final logistic regression model and the *y* axis is the actual probabilities. The long-dashed line represents an ideal nomogram whose predicted outcome perfectly corresponds to the actual outcome. The solid line (bias-corrected) represents the bootstrap-corrected performance of our nomogram, and the short-dashed line represents apparent accuracy of the nomogram. The apparent and bias-corrected line fell approximately along the ideal line, which indicates that the calculated by the nomogram accurately represents the actual prediction of pathological complete response (pCR) for rectal cancer in both the primary and validation cohorts.

The results of multivariate logistic regression are shown in Table 2. The analysis revealed that patients with a negative nodal stage (odds ratio (OR) = 3.2, 95% confidence interval (CI) = 1.279–8.410, *P*=0.0135), with high Hb levels (>10 g/dL) during neoadjuvant CRT (OR = 3.067, 95% CI = 1.251–8.187, *P*=0.0125), receiving FOLFOX-based neoadjuvant CRT (OR = 5.385, 95% CI = 1.699–17.688, *P*=0.0044), with a long interval (>8 weeks) between radiotherapy and surgery (OR = 1.135, 95% CI = 1.021–5.712, *P*=0.0469), and with post-CRT CEA levels ≤2 ng/mL (OR = 2.891, 95% CI = 1.156–7.369, *P*=0.0233) were more likely to achieve a pCR. In addition, we compared a combination of FOLFOX-based neoadjuvant CRT and a longer interval of >8 weeks with a combination of fluoropyrimidine-based neoadjuvant CRT and a shorter interval of ≤8 weeks; FOLFOX regimen plus a longer interval was revealed to be an independent predictor of a pCR (OR, 5.518, 95% CI = 1.826–18.261, *P*=0.0024). Therefore, the prediction nomogram (Figure 1) was constructed to predict pCR by incorporating the four significant predictors: (I) FOLFOX-based neoadjuvant CRT with a longer interval of >8 weeks; (II) Hb level during neoadjuvant CRT; (III) post-CRT CEA level; and (IV) clinical N stage. Each predictor was assigned a score on the point scale. We could draw a vertical line downwards from the total point to obtain the probability of pCR rates. The C-index was 0.74 (95% confidence interval 0.65–0.84), which was used to test the discriminative ability of the model. The goodness-of-fit of the model was evaluated by the calibration curve (Figure 2), which revealed that the predicted probabilities of pCR fit well to the observed probabilities of pCR.

**Figure 3:**
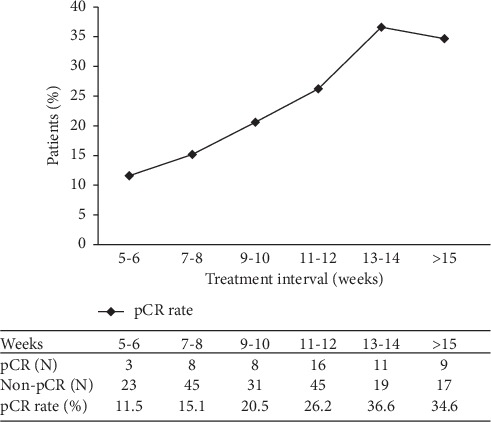
Percentage of patients with a pathological complete response (pCR) at different intervals between radiotherapy and surgery.

Figure 3 shows the percentage of patients with a pCR after different intervals between radiotherapy and surgery. Overall, the pCR rate increased as the radiotherapy-surgery interval was prolonged from 11.5% (5–6 week interval) to 36.6% (>13 week interval). The pCR rate reached a plateau after an interval of >13 weeks.

### 3.1. Patient Outcomes

**Figure 4:**
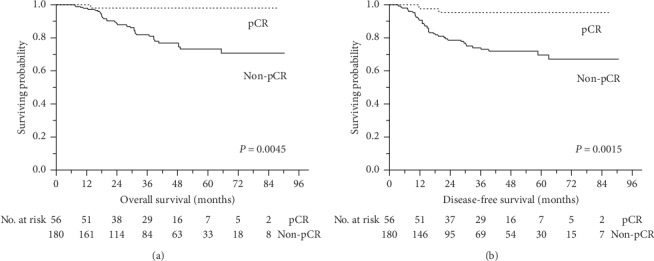
Overall and disease-free survival in patients with locally advanced rectal cancer. Overall survival (a) and disease-free survival (b) between patients with and without a pathological complete response (pCR).

The median follow-up time was 36 months (range, 6–90 months). In this cohort, 35 of 236 patients (14.8%) died. The estimated 3- and 5-year OS rates for all patients were 85.4% and 78.1%, respectively. Patients who achieved a pCR had greater survival than those who did not (Figure 4(a); *P*=0.0045). The 3- and 5-year OS rates were both 97.9% for patients with a pCR, whereas they were 81.7% and 72.9%, respectively, for patients without a pCR. The 3- and 5-year DFS rates for all patients were 79.3% and 75.5%, respectively. Patients with a pCR had higher DFS than those without a pCR (Figure 4(b); *P*=0.0015). The 3- and 5-year DFS were both 95.5% for the pCR group, whereas they were 74.3% and 69.7%, respectively, for the non-pCR group.

### 3.2. Failure Patterns

Table 3 shows that 2 patients (3.6%) and 42 patients (23.3%) from the pCR and non-pCR groups, respectively, developed tumor recurrence. In the pCR group, one patient developed local recurrence and bilateral adrenal metastases at 7 months after neoadjuvant CRT completion. Although FOLFIRI plus bevacizumab was administrated, she died of cancer progression at 3 months after the introduction of FOLFIRI plus bevacizumab. The other pCR patient had liver metastases at 15 months after neoadjuvant CRT. He received FOLFIRI plus bevacizumab, and tumor remained under control for over 8 months after liver metastases. In the non-pCR group, the risk of distant metastasis (13.2%) was higher than that of locoregional recurrence (6.7%). The most common sites for distant metastases in the non-pCR group were the liver (*n* = 15), lung (*n* = 12), and peritoneum (*n* = 4).

## 4. Discussion

For patients with LARC following neoadjuvant CRT, patients achieving a pCR have had higher survival rates than those who did not [5, 21]. In this study, patients who achieved a pCR had a better OS and DFS compared with those who did not. We found that patients with a clinically negative nodal disease, without anemia during treatment, with a long interval (>8 weeks) between radiotherapy and surgery, with post-CRT CEA levels ≤2 ng/mL, and receiving a FOLFOX-based regimen were more likely to achieve a pCR to CRT. Furthermore, patients who received a FOLFOX-based regimen plus a long interval (>8 weeks) between radiotherapy and surgery achieved a high pCR rate, which was consistent with that of our previous study [5]. We previously reported that extending FOLFOX and prolonging the radiotherapy-surgery interval resulted in a high pCR rate (31.6%) for patients with LARC undergoing neoadjuvant CRT.

We reported a higher pCR rate compared with that reported by the German rectal trial (22.5% vs. 8%) [1]. This improvement in the pCR rate might be because of biweekly FOLFOX administration during and after the whole course of irradiation in our cohort compared with bolus 5-FU only during radiotherapy in the German rectal trial. In this study, patients receiving FOLFOX-based neoadjuvant CRT had a significantly higher pCR rate than those undergoing fluoropyrimidine-based neoadjuvant CRT (27.2% vs. 16.7%; *P*=0.0411). This is supported by the results of the German CAO/ARO/AIO-04 trial, which showed pCR rates of 17% and 13% (OR, 1.40; 95% CI, 1.02–1.92; *P*=0.038) in the 5-FU plus oxaliplatin group and fluorouracil group, respectively [22]. Another reason for a higher pCR rate in our cohort might be that most patients (88.9%) in the fluoropyrimidine-based neoadjuvant CRT group received continuous capecitabine. Patients receiving 5-FU or capecitabine continuously during radiotherapy have shown improved pCR rates compared with those receiving bolus 5-FU [4, 15]. Therefore, our overall pCR rate was higher than that reported in the German rectal trial.

Clinical T stages, radiation doses, and pre-CRT serum CEA levels were not predictive factors of a pCR in the current study, although some studies have shown that pretreatment CEA level was a crucial pCR predictor [12, 14]. Furthermore, a smaller tumor size and a higher radiation dose have been reported to be associated with increased pCR rates [12, 13, 16].

In colorectal cancer (CRC), positive pathological nodes have been associated with poor prognosis [13, 21, 23]. For patients with LARC following neoadjuvant CRT, pretreatment negative lymph nodes have been correlated with increased pCR rates [13, 21]. Clinical N stage is not always correlated with pathological N stage [24]. It might not be easy to differentiate reactive nodes from metastatic nodes in current image studies. However, positive pretreatment nodal status usually represents tumor progression and aggression. In our cohort, 39.4% of the patients with clinically negative N stage achieved a pCR, whereas only 19.3% of the patients with pretreatment positive N stage achieved a pCR. Therefore, clinical non-N stage was considered to be a potential indicator of pCR in our study.

Hb levels have been associated with oncologic outcomes in numerous malignancies including head and neck cancer, cervical cancer, and CRC [25–27]. In general, anemia is defined as Hb level of ≤12 g/dL in women and ≤13.5 g/dL in men according to the Third National Health and Nutrition Examination Survey [28]. However, we defined anemia as Hb level of ≤10 g/dL according to the CTCAE 4.03 because we attempted to correlate treatment toxicity with pCR achievement. The role of pretreatment anemia in treatment response and tumor control has been reported [27, 29]. Berardi et al. identified that Hb level of >12 g/dL was associated with higher DFS and tumor downstaging for patients with rectal cancer receiving neoadjuvant radiotherapy with or without chemotherapy in a retrospective review of 317 patients [29]. Khan et al. studied clinical parameters of 463 patients with LARC and found that a pretreatment Hb level of >12 g/dL was associated with response to neoadjuvant CRT and risk of local recurrence [30]. Our study found that Hb level of >10 g/dL during neoadjuvant CRT was a significant predictor of increased pCR rates. To our knowledge, this is the first study to correlate treatment-related anemia with probabilities of pCR to neoadjuvant CRT in LARC.

In general, severe toxicity might reflect favorable treatment response except for achievement rates. However, patients with low Hb levels during neoadjuvant CRT experienced unfavorable treatment response in our study. Because anemia has been correlated to tumor hypoxia, angiogenesis, and resistance to chemotherapy and radiotherapy [7, 31], we suggested that high Hb levels during treatment could sensitize tumor cells to radiation due to improved oxygenation. This might explain why severe hematologic toxicity did not translate into better treatment response.

We found patients receiving FOLFOX-based neoadjuvant CRT to be more likely to achieve a pCR compared with those receiving fluoropyrimidine-based neoadjuvant CRT (27.2% vs. 16.7%; *P*=0.0411). Our results are supported by 2 phase III randomized studies. The phase III German CAO/ARO/AIO-04 trial demonstrated that addition of oxaliplatin in a neoadjuvant therapy improved the pCR rate and DFS compared with fluorouracil-based neoadjuvant CRT [22, 32]. Another phase III Chinese FOWARC trial studied the combination of fluorouracil-based neoadjuvant CRT with and without oxaliplatin for patients with LARC and demonstrated that FOLFOX-based neoadjuvant CRT resulted in a higher pCR rate than 5-FU-based neoadjuvant CRT [7]. However, the effectiveness of oxaliplatin addition to fluoropyrimidine-based neoadjuvant CRT remains undetermined because some phase III trials have shown similar pCR rates between oxaliplatin-containing and fluoropyrimidine-based neoadjuvant CRT [6, 31]. This discrepancy among those trials might be because of various dose accumulation of oxaliplatin in each trial, the different treatment duration of delivering oxaliplatin during irradiation or after completion of irradiation, and possibly different treatment response between patients of different ethnicities.

The interval between radiotherapy and surgery has been associated with a pCR to neoadjuvant CRT [33, 34]. Intentional prolongation of the interval between radiotherapy and surgery has been investigated in 2 randomized studies. The Lyon R90-01 trial compared outcomes for intervals of 2 and 6 to 8 weeks between radiotherapy and surgery and demonstrated that a longer interval resulted in favorable pathological downstaging [35]. The Istanbul R-01 trial investigated the outcomes of surgery at 4 and 8 weeks after neoadjuvant CRT and found no difference in tumor regression between the 2 intervals [36]. However, we observed a pCR rate of >33% at an interval of 13 weeks. A longer interval (>8 weeks) seemed to be associated with a higher chance of pCR. This is supported by several retrospective studies [5, 33, 37]. Sloothaak et al. reviewed 1593 patients with LARC and found that delaying surgery until the 10th to 11th week after the end of radiotherapy achieved the highest pCR rate [34]. Rombouts et al. studied 1073 patients with LARC and revealed that radiotherapy-surgery intervals of 9 to 12 weeks resulted in a high pCR rate [37].

Some studies have reported that post-CRT clinical factors were associated with pCR [23, 38]. In the current study, several post-CRT variables were analyzed and post-CRT CEA ≤2 ng/mL was an independent predictor of increased pCR rates. CEA is associated with prognosis and treatment response in patients with LARC treated with neoadjuvant CRT [39]. Deceased post-CRT CEA levels have been reported to be associated with increased pCR rates in LARC patients following neoadjuvant CRT [38, 40]. Lowering post-CRT CEA levels might imply decreased tumor burdens and therefore, a favorable response to CRT was achieved.

We established the prediction nomogram model to predict a pCR for LARC patients following neoadjuvant CRT. FOLFOX-based neoadjuvant CRT with a longer interval of >8 weeks, Hb > 10 g/dL during neoadjuvant CRT, a clinical N0 stage, and post-CRT CEA ≤2 ng/mL were significant predictors of increased pCR rates. This data could facilitate patient selection and help clinicians to identify the right patients who may benefit most from watch-and-wait strategies. However, validating the prediction nomogram on an independent cohort would be mandatory. Although we did not have an independent cohort for external validation of the nomogram, we carried out a bootstrap method for internal validation of our model. The C-index of our model was 0.74, which indicated that our nomogram had a high predictive accuracy of 74%. Thus, our model wound be helpful in clinical use.

This study had several limitations. First, this was a retrospective study, and heterogeneity in patient selection and treatment decision by individual physicians may have influenced the results. Second, we only reported a correlation between Hb levels and a pCR, but other associated inflammation-based parameters, such as serum albumin, C-reactive protein, and neutrophil and lymphocyte counts, were not completely collected. These inflammation-related factors have been reported to be associated with tumor response to neoadjuvant CRT for LARC [41, 42]. Third, MRI features were not included for analysis due to some missing data, though evidence has shown MRI features to be predictive factors of pCR in LARC patients [43, 44].

## 5. Conclusions

Our results demonstrated that clinical negative nodal diseases, Hb levels >10 g/dL during neoadjuvant CRT, FOLFOX-based neoadjuvant CRT with a longer radiotherapy–surgery interval, and post-CRT CEA levels ≤ 2 ng/mL resulted in a greater likelihood of a pCR. The nomogram model to predict a pCR rate for LARC patients following neoadjuvant CRT would be potential in clinical implication; however, further prospective, randomized large-scale studies are warranted to validate our results.

## Figures and Tables

**Table 1:** Clinical characteristics of patients with locally advanced rectal cancer after trimodality treatment.

	pCR (%)	Non-pCR (%)	*P* value^*∗*^
All patients, no.	56 (23.7)	180 (76.3)	—
Gender			0.4139
Male	34 (22.1)	120 (77.9)	
Female	22 (26.8)	60 (73.2)	
Age at diagnosis			0.3590
≤60	21 (20.8)	80 (79.2)	
>60	35 (25.9)	100 (74.1)	
Location of tumor			0.1564
Upper	18 (19.0)	77 (81.0)	
Middle/low	38 (27.0)	103 (73.0)	
Grade			0.0577
Well differentiated	0 (0)	16 (100)	
Moderate differentiated	53 (25.0)	159 (75.0)	
Poor differentiated	3 (37.5)	5 (62.5)	
Clinical T stage			0.1964
T2	4 (30.8)	9 (69.2)	
T3	47 (25.5)	137 (74.5)	
T4	5 (12.8)	34 (87.2)	
Clinical N stage			0.0202
N0	14 (39.0)	22 (61.0)	
N+	42 (21.0)	158 (79.0)	
Radiation dose (cGy)			0.0772
≤4500	5 (11.4)	39 (88.6)	
>5000	51 (88.6)	141 (11.4)	
Chemotherapy			
FOLFOX	40 (28.4)	101 (71.6)	0.0412
Fluoropyridine	16 (16.8)	79 (83.2)	
Cycles of pre-OP chemotherapy			0.5215
<7	39 (25)	117 (75)	
≥7	17 (21.3)	63 (78.7)	
Pre-CRT CEA (ng/mL)			0.0674
≤5	40 (27.8)	104 (72.2)	
>5	16 (17.4)	76 (82.6)	
Anemia during CRT			0.0159
Hb (g/dL) > 10	47 (28.0)	121 (72.0)	
Hb (g/dL) ≤ 10	9 (13.2)	59 (86.8)	
Leukopenia during CRT			0.1113
WBC > 3000 (/*μ*L)	29 (20.3)	114 (79.7)	
WBC ≤ 3000 (/*μ*L)	27 (29.4)	65 (70.6)	
RT to surgery interval			0.0081
≤8 weeks	11 (13.6)	70 (86.4)	
>8 weeks	45 (29.0)	110 (71.0)	
ycT stage			0.0734
T0	4 (33.3)	8 (66.7)	
T1	1 (11.1)	9 (88.9)	
T2	8 (32)	17 (68)	
T3	35 (26.1)	99 (73.9)	
T4	8 (14.3)	48 (85.7)	
ycN stage			0.1515
N0	30 (20.5)	116 (79.5)	
N+	26 (28.9)	64 (71.1)	
Post-CRT CEA (ng/mL)			0.0285
≤2	9 (45)	11 (55)	
>2	47 (21.8)	169 (78.2)	

CEA, carcinoembryonic antigen; CRT, chemoradiation therapy; FOLFOX, fluorouracil, leucovorin, and oxaliplatin; Hb, hemoglobin; OP, operative; pCR, pathological complete response; RT, radiation therapy; WBC, white blood cell; ycT stage: clinical tumor stage after chemoradiotherapy; ycN stage: clinical nodal stage after chemoradiotherapy. ^*∗*^Fisher's exact test.

**Table 2:** Independent clinical parameters significantly associated with a pCR.

Parameter	OR	95% CI	*P* value^*∗*^
Chemotherapy (FOLFOX vs. non-FOLFOX)	5.385	1.699–17.688	0.0044
Clinical N stage (N0 vs. N1/2)	3.200	1.279–8.410	0.0135
Anemia during CRT (Hb > 10 vs. Hb≤ 10)	3.067	1.251–8.187	0.0125
Post-CRT CEA (≤2 vs. > 2)	2.891	1.156–7.369	0.0233
RT to surgery interval (>8 weeks vs. ≤8 weeks)	1.135	1.021–5.712	0.0469
Gender (male vs. female)	0.607	0.291–1.265	0.1823
Age (≤60 vs. > 60)	0.680	0.329–1.265	0.2850
Location of tumor (middle/low vs. upper)	1.895	0.911–4.068	0.0877
Grade (WD/MD vs. PD)	0.349	0.064–2.113	0.2373
Clinical T stage (T2/3 vs. T4)	1.810	0.615–6.186	0.2907
Radiation dose (<5000 vs. ≥ 5000)	0.626	0.186–1.799	0.3972
Pre-CRT CEA (≤5 vs. > 5)	1.761	0.861–3.727	0.1221
Leukopenia during CRT(WBC>3000 vs. WBC≤3000)	0.833	0.378–1.826	0.6479
Cycles of pre-OP chemotherapy(≥7 vs. < 7)	1.037	0.656–1.625	0.8732
ycT stage (T0-2 vs. T3-4)	1.872	0.886–4.110	0.1008
ycN stage (N0 vs. N1/2)	1.624	0.799–3.311	0.1788

CEA, carcinoembryonic antigen; CRT, chemoradiation therapy; FOLFOX, fluorouracil, leucovorin, and oxaliplatin; Hb, hemoglobin; OP, operative; pCR, pathological complete response; RT, radiation therapy; WBC, white blood cell; ycT stage: clinical tumor stage after chemoradiotherapy; ycN stage: clinical nodal stage after chemoradiotherapy. ^*∗*^Logistic regression.

**Table 3:** Failure patterns among patients with locally advanced rectal cancer after trimodality treatment.

Recurrence	pCR	Non-pCR
Local/regional only	0	12/180 (6.7%)
Distant only	1/56 (1.8)	24/180 (13.2%)
Local/regional/distant	1/56 (1.8)	6/180 (3.3%)
Total	2/56 (3.6)	42/180 (23.3%)

pCR, pathological complete response.

## Data Availability

The data used to support the findings of this study are included within the article, and the data sources are available from the corresponding author upon request.
